# Method for estimating discharge, hydraulic depth, and mean velocity in rivers through spatial interpolation of at-a-station hydraulic geometry in data- scarce regions^✰^^✰✰^

**DOI:** 10.1016/j.mex.2026.103804

**Published:** 2026-01-24

**Authors:** Eduardo Zamudio-Huertas, César Augusto García-Ubaque, Nelson Obregón-Neira

**Affiliations:** a Assistant Professor Universidad Distrital Francisco José de Caldas, Bogotá, Colombia; b Titular Professor Universidad Distrital Francisco José de Caldas, Bogotá, Colombia; c Titular Professor Pontificia Universidad Javeriana, Bogotá, Colombia

**Keywords:** Discharge calculation, River width, Hydraulic geometry, Interpolation, Multiquadric

## Abstract

Reliable discharge estimation is essential for water resource management, yet many regions lack sufficient hydrological stations. To address this limitation, we propose the Spatial Hydraulic Geometry Interpolation (SHGI) method, which estimates discharge (Q), hydraulic depth (D), and mean velocity (V) from river width (W) obtained via surveys or satellite imagery. SHGI integrates hydraulic geometry theory with multiquadric radial basis interpolation, applied to the Meta and Atrato river basins in Colombia. Parameters of at‑station hydraulic geometry (coefficients a, *c, k* and exponents *b, f, m*) were derived using least squares and transformed into log‑ratio space to preserve their compositional constraints. Interpolation along upstream distance ensures spatial continuity, and closure operations guarantee internal consistency. Validation against observed data in basins with contrasting geomorphology and data density confirmed the method’s robustness.

The principal contributions of SHGI are:

• Longitudinal continuity: explicit incorporation of upstream distance to interpolate parameters consistently along channels and tributaries.
• Compositional integrity: preservation of the multiplicative and additive constraints of hydraulic geometry parameters during interpolation.
• Estimation under data scarcity: enabling calculation of Q, D, and V at ungauged sites using only river width.

## Graphical abstract

**Figure:**
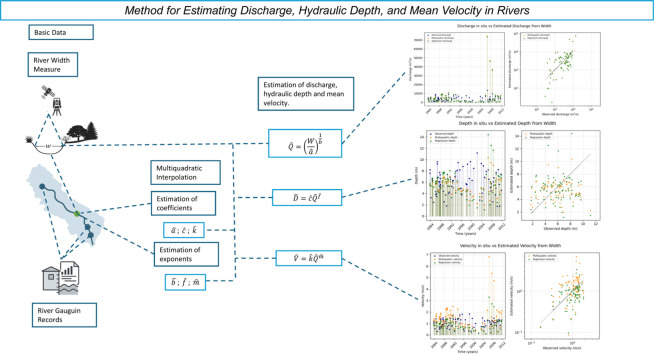

## Specifications table

**Table:** 

**Subject area**	Engineering
**More specific subject area**	*River hydraulics*
**Name of your method**	*Spatial Hydraulic Geometry Interpolation - SHGI*
**Name and reference of original method**	*N/A*
**Resource availability**	*N/A*

## Background

Water resource planning and management rely on national policies aimed at ensuring sustainability, adequate supply, ecosystem conservation, and risk mitigation. Achieving these objectives requires reliable technical information, particularly on river discharge, which is traditionally obtained from historical records at hydrological stations. However, in many regions with limited infrastructure or difficult access, the absence of discharge data poses a critical barrier to informed decision-making.

To address this limitation, it is necessary to develop methods that estimate hydraulic variables such as discharge, hydraulic depth, and average velocity in areas without direct measurements. In this context, the hydraulic geometry framework proposed by [1] provides an empirical basis for relating discharge to geometric characteristics of the river cross-section: the surface width (W), hydraulic depth (D), and a hydraulic variable velocity (V) to discharge (Q) through the following empirical power-law equations:

(1) $$\begin{array}{c} W=aQ^{b}\\ D=cQ^{f}\\ V=kQ^{m} \end{array}$$

Where, the coefficients a, c, k and the exponents b, f, m are parameters to be determined from the empirical model, the above equations. The continuity equation is satisfied for any cross-section of a river, as follows:

(2) $$Q=AV=W\frac{A}{W}V=WDV$$

By replacing equations (1) in the continuity Eq. (2) we obtain:

(3) $$Q=aQ^{b}cQ^{f}kQ^{m}$$

To satisfy the equality in Eq. (3) the product of the coefficients and the sum of the exponents must be equal to 1.

(4) $$\begin{array}{c} ack=1\\ b+f+m=1 \end{array}$$

Gleason & Smith [2] proposed representing the coefficients and exponents using semi-logarithmic diagrams, observing a linear trend that allows the coefficients to be estimated from the exponents. This approach, known as multi-station hydraulic geometry, has been successfully applied by Du et al [3] and Yuan et al [4] to estimate discharge from river width measured with satellite imagery.

Kratzer et al [5] evaluated interpolation methods for distributed hydraulic variables and concluded that the LOESS method, with a smoothing parameter between 0.2 and 0.4, provides interpolations with low uncertainty. Combining these methodologies improves the estimation of hydraulic variables in areas with scarce information, although limitations remain.

Building on these approaches, the present study introduces the Spatial Hydraulic Geometry Interpolation (SHGI) method, which ensures spatial continuity along the main channel and its tributaries when estimating discharge (Q), hydraulic depth (D), and mean velocity (V) based on hydraulic geometry at station scale, while preserving their compositional nature [6]. SHGI achieves this by interpolating hydraulic geometry coefficients and exponents using a multiquadric radial basis function [7], particularly suitable for parameters exhibiting high variability especially the coefficients, which have been less studied than the exponents due to their dependence on measurement units [8] and their complex functional forms [9]. By integrating these elements, SHGI provides a robust framework for estimating hydraulic variables in data-scarce regions, overcoming limitations observed in previous approaches focused solely on discharge.

## Method details

To ensure reproducibility and clarity, the proposed method (SHGI) is formalized using mathematical notation and sequential steps. The approach is based on steady-state hydraulic geometry theory, which relates discharge (Q) to width (W), hydraulic depth (D), and average velocity (V) of the channel using power law functions.

In this study, these relationships are extended by interpolating hydraulic geometry parameters throughout the river network using radial basis functions, a technique that is ideal for variables with high spatial variability. This formulation preserves the compositional nature of the parameters (the product of the coefficients equals one and the sum of the exponents equals one) and ensures spatial continuity along the main channel and its tributaries. To ensure reproducibility and clarity, the method (SHGI) is presented using mathematical notation and sequential steps, as described below:

1. Identify the hydrological stations located upstream and downstream of the target site for estimating hydraulic variables: discharge (Q), depth (D), and average velocity (V).

2. Retrieve historical streamflow records from all hydrological stations in the selected network.

3. Using the historical discharge records at each of the selected hydrological stations, estimate the coefficients and exponents of the empirical power-law equations for the station's hydraulic geometry (Equations 1) using the least squares method, following the procedure described by [10]. Table 1 summarizes the corresponding equations for each coefficient and exponent.

**Table 1:** Least squares estimation formulas for hydraulic geometry parameters.

Parameter	Least squares magnitude
a	$a=\frac{\bar{W}}{{\bar{Q}}^{b}}$
b	$b=\frac{\sum \left(lnQ_{i}-\bar{lnQ}\right)\left(lnW_{i}-\bar{lnW}\right)}{\sum {\left(lnQ_{i}-\bar{lnQ}\right)}^{2}}$
c	$c=\frac{\bar{D}}{{\bar{Q}}^{f}}$
f	$f=\frac{\sum \left(lnQ_{i}-\bar{lnQ}\right)\left(lnD_{i}-\bar{lnD}\right)}{\sum {\left(lnQ_{i}-\bar{lnQ}\right)}^{2}}$
k	$k=\frac{\bar{V}}{{\bar{Q}}^{m}}$
m	$m=\frac{\sum \left(lnQ_{i}-\bar{lnQ}\right)\left(lnV_{i}-\bar{lnV}\right)}{\sum {\left(lnQ_{i}-\bar{lnQ}\right)}^{2}}$

Formulas adapted from [10], “Generalized Linear Models.”.

4. Since the parameters of station hydraulic geometry calculated at each of the hydrological stations are compositional in nature, as they satisfy the constraints of equations (5) that link them, i.e., they are not independent, it is necessary to perform logarithmic transformations (Aitchison space) to avoid inconsistent results or violations of compositional constraints. The most common transformation is the centered log-ratio (CLR) transformation [11], defined as:

For the coefficients (a, c, k):

(5) $$clr\left(a,c,k\right)=\left[log\left(\frac{a}{g}\right),\;log\left(\frac{c}{g}\right),\;log\left(\frac{k}{g}\right)\right],\;g={\left(ack\right)}^{\frac{1}{3}}$$

For the exponents (b, f, m)

(6) $$clr\left(b,f,m\right)=\left[log\left(\frac{b}{g}\right),\;log\left(\frac{f}{g}\right),\;log\left(\frac{m}{g}\right)\right],\;g={\left(bfm\right)}^{\frac{1}{3}}$$

5. In Aitchison's space, the coefficients and exponents are interpolated at the point of interest using a multiquadratic radial basis function, defined by the following expression:

(7) $$P\left(d\right)=\sum_{i}^{n}\lambda_{i}\sqrt{∥d-d_{i}{∥}^{2}+\alpha^{2}}$$

where:

P represents each parameter to be estimated ($\hat{a}$, $\hat{b}$, $\hat{c}$, $\hat{f}$, $\hat{k}$, $\hat{m}$),
di are the distances upstream from each hydrological station,
λi are the weights are estimated from a distance matrix and the vector P of known parameters.
The inverse transformation is then applied, ensuring compositional constraints.

6. Once the parameters have been interpolated in the logarithmic space, the closures are applied to ensure the compositional constraints (equations: 4 and 5) of the hydraulic geometry at the station using the following equations:

Multiplicative closure (coefficients $\hat{a},\;\hat{c},\;\hat{k}$)

(8) $$V^{*}=\text{exp}[log\left(V\right)-\bar{log\left(V\right)}$$

Where:

*V* = [$\hat{a},\;\hat{c},\;\hat{k}]$ is the vector of the estimated parameters and $\bar{log(V)}$ is the average of the logarithms of the vector V.
This closure guarantees that: $\hat{a}*\hat{c}*\;\hat{k}=1$.
Additive closure (exponents $\hat{b},\;\hat{f},\;\hat{m}$)
  (9) $$U^{*}=\frac{U}{\sum U}$$

Where:

***U*** = [$\hat{b},\;\hat{f},\;\hat{m}]$ is the vector of the estimated exponents and ∑U the sum of the estimated exponents of the vector **U.** This closure guarantees that: $\hat{b}+\;\hat{f}+\;\hat{m}=1$.

7. At points of interest where information is limited or scarce, measure the width of the river (W) using a topographic survey or remote sensors and estimate the discharge ($\hat{Q}$), hydraulic depth ($\hat{D}$) and average velocity ($\hat{V}$), using the following equations:

(10) $$\begin{array}{c} \hat{Q}={\left(\frac{W}{\hat{a}}\right)}^{1\;/\;\hat{b}}\\ \hat{D}=\hat{c}\cdot {\hat{Q}}^{\hat{f}}\\ \hat{V}=\hat{k}\cdot {\hat{Q}}^{\hat{m}} \end{array}$$

The above procedure is summarized in the following pseudo-code: (Algorithm 1)

**Algorithm 1:** Spatial Interpolation of Hydraulic Geometry at-Station Parameters via Multiquadric.

**Input:** River width W, upstream distance d_i_, station data {a_i_, b_i_, c_i_, f_i_, k_i_, m_i_}
**Output:** Estimated $\hat{Q}$, $\hat{D}$, $\hat{V}$
**Step 1:** Collect historical discharge records from upstream and downstream stations.
**Step 2:** Estimate hydraulic geometry parameters (a,b,c,f,k,m) using least squares based on Table 1.
**Step 3:** Apply log-ratio transformation to preserve compositional constraints based on Eqs. (5) and (6).
**Step 4:** Interpolate parameters along river using multiquadric interpolation based on Eq. (7):
Choose an optimal shape parameter α
For each parameter p in {a,b,c,f,k,m}:
p_est(d) = MQI(p, stations, d)
**Step 5:** Close the estimated coefficients and exponents to ensure compositional constraints based on
Eqs. (8) and (9)
**Step 6:** Compute $\hat{Q}$, $\hat{D}$, $\hat{V}$ based on Eqs. (10)
**Step 7:** Return $\hat{Q}$, $\hat{D}$, $\hat{V}$

Algorithm adapted from the proposed SHGI methodology described in this study. Steps follow the interpolation procedure based on multiquadric radial basis functions [7].

## Method validation

To validate the proposed method (SHGI) and assess its robustness under contrasting hydrological conditions, the procedure was applied to two river basins in Colombia: the Atrato and Meta rivers. These basins differ significantly in geomorphology and discharge regime, providing an appropriate framework to evaluate the adaptability and accuracy of the approach. According to the Institute of Hydrology, Meteorology and Environmental Studies (Ideam), the Atrato River has an average annual discharge of 2550 m³/s, while the Meta River reaches 6614 m³/s [12]. This contrast allows testing the method under diverse hydraulic and geographical settings, ensuring its applicability in data-scarce regions with varying environmental characteristics. Additionally, the density of hydrological stations also reflects this contrast: 13 stations were analyzed in the Atrato basin, compared to 48 stations in the Meta basin, offering different levels of spatial data availability for validation.

Streamflow data from 13 stations in the Atrato River basin and 46 hydrological stations in the Meta River basin, all operated by Ideam, were collected and analyzed. In the Meta River basin, 18 stations are located along the main channel and the remaining 28 on its tributaries; in the Atrato River basin, eight stations are on the main channel and five on tributaries. Using this information, at-station hydraulic geometry parameters were estimated through the least squares method, and the upstream distance was calculated for each hydrological station in both basins.

**Fig. 1:**
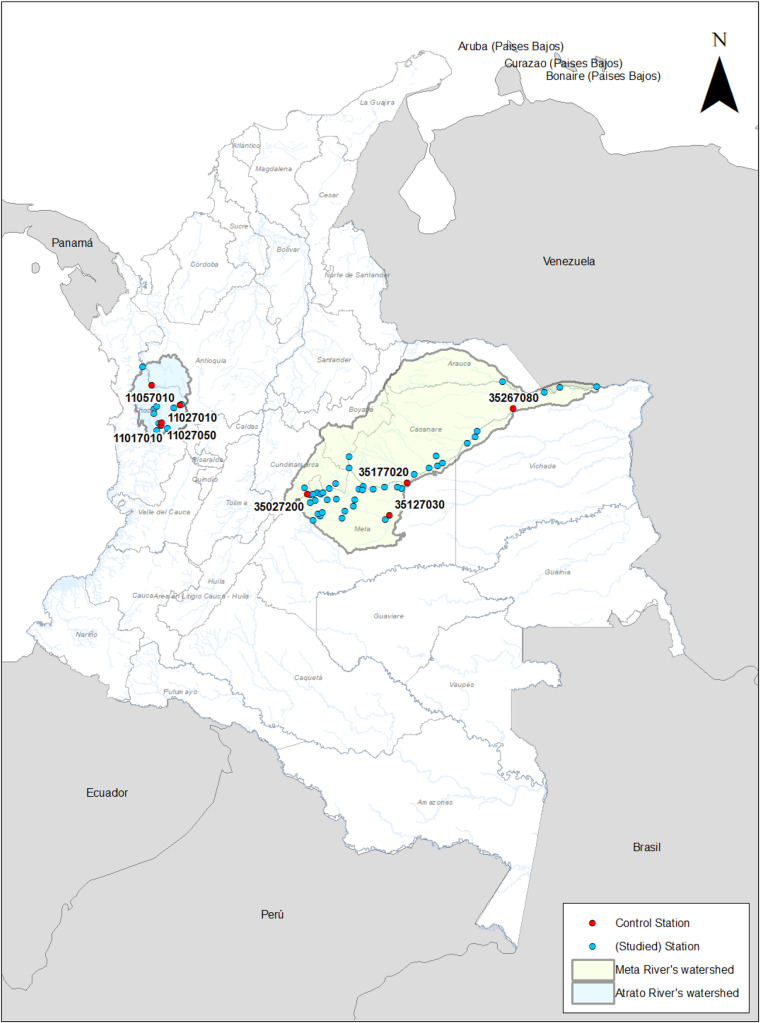
Spatial distribution of hydrological stations in the Atrato and Meta river basins. Spatial distribution of gauging stations included in the analysis. Red dots represent control stations, while blue dots correspond to stations with available discharge, width, hydraulic depth, and mean velocity records within the Atrato and Meta river basins.

The geographic information of the selected hydrological stations, together with the upstream distance and the hydraulic geometry parameters estimated using the least squares method, is summarized in Table 2, Table 3. The spatial location of these stations is shown in Fig. 1.

**Table 2:** Geographic information, upstream distance, and at-station hydraulic geometry parameters for hydrological stations in the Meta River basin.

Hydrological station code	latitude	longitude	a	b	c	f	k	m	Upstream [m]	Group river
35,017,020	4.10	−72.94	34.16	0.22	0.36	0.37	0.08	0.41	573.50	Meta
35,017,030	3.80	−73.58	7.31	0.61	0.28	0.26	0.48	0.13	45.16	Meta
35,017,100	3.98	−72.97	86.69	0.10	0.11	0.53	0.10	0.38	553.88	Meta
35,107,030	4.28	−72.79	73.65	0.11	0.34	0.43	0.04	0.47	1067.56	Meta
35,117,010	4.33	−72.39	175.04	0.04	0.40	0.36	0.01	0.60	1249.62	Meta
35,117,020	4.30	−72.60	45.94	0.26	0.13	0.46	0.16	0.27	1216.45	Meta
35,177,010	4.73	−71.40	114.93	0.21	0.20	0.38	0.04	0.40	1753.85	Meta
35,177,020	4.42	−71.96	116.19	0.15	0.20	0.43	0.04	0.43	1675.17	Meta
35,207,010	4.57	−71.83	42.79	0.30	0.14	0.46	0.17	0.24	1697.53	Meta
35,217,020	4.69	−71.56	25.72	0.33	0.29	0.34	0.13	0.34	1749.47	Meta
35,227,060	4.78	−71.32	2.28	0.68	1.08	0.17	0.41	0.14	1754.61	Meta
35,257,010	6.08	−69.42	11.91	0.50	7.93	0.04	0.01	0.54	2513.54	Meta
35,257,020	6.18	−69.13	1.45	0.74	2.5	0.09	0.28	0.17	2555.67	Meta
35,257,040	6.18	−68.44	23.76	0.44	1.01	0.19	0.04	0.37	2640.66	Meta
35,267,020	5.15	−70.85	78.6	0.24	0.26	0.37	0.05	0.39	1938.65	Meta
35,267,030	5.27	−70.71	30.62	0.37	0.79	0.22	0.04	0.40	1959.75	Meta
35,267,080	5.79	−69.99	57.41	0.28	0.50	0.30	0.03	0.42	2064.00	Meta
35,267,100	5.37	−70.67	108.31	0.19	0.63	0.28	0.01	0.53	1971.19	Meta
35,017,040	3.75	−73.18	16.18	0.29	0.32	0.39	0.19	0.32	235.55	Tributary
35,017,050	3.88	−73.12	48.73	0.13	0.15	0.54	0.14	0.33	357.78	Tributary
35,017,070	3.86	−73.54	15.47	0.12	0.36	0.31	0.18	0.58	38.44	Tributary
35,017,080	3.83	−73.62	15.61	0.18	0.19	0.41	0.34	0.41	20.50	Tributary
35,017,090	3.71	−73.71	6.64	0.25	0.41	0.33	0.36	0.42	6.07	Tributary
35,027,140	4.05	−73.76	18.69	0.20	0.23	0.36	0.23	0.44	86.42	Tributary
35,027,160	4.20	−73.72	5.93	0.44	0.30	0.31	0.57	0.25	66.60	Tributary
35,027,180	4.10	−73.45	6.59	0.19	0.40	0.34	0.38	0.47	32.39	Tributary
35,027,190	4.31	−73.87	14.59	0.10	0.27	0.44	0.25	0.46	40.64	Tributary
35,027,200	4.21	−73.82	20.55	0.11	0.21	0.38	0.23	0.51	54.74	Tributary
35,027,210	4.20	−73.77	26.84	0.04	0.19	0.46	0.20	0.50	60.43	Tributary
35,037,100	4.24	−73.64	11.79	0.31	0.25	0.30	0.34	0.38	26.60	Tributary
35,037,110	4.23	−73.53	6.34	0.29	0.46	0.44	0.34	0.28	10.76	Tributary
35,037,130	4.09	−73.67	18.11	0.23	0.21	0.35	0.27	0.43	5.89	Tributary
35,037,140	4.12	−73.28	12.27	0.24	0.28	0.42	0.29	0.34	52.69	Tributary
35,047,030	4.31	−73.41	17.52	0.27	0.27	0.35	0.21	0.39	25.90	Tributary
35,057,010	4.40	−73.29	30.75	0.18	0.37	0.30	0.09	0.52	23.12	Tributary
35,057,040	4.20	−73.57	7.89	0.18	0.23	0.20	0.54	0.62	11.58	Tributary
35,097,090	4.90	−73.05	28.24	0.17	0.25	0.30	0.14	0.53	27.33	Tributary
35,107,010	4.30	−72.87	20.40	0.19	0.30	0.48	0.16	0.33	56.48	Tributary
35,107,020	4.35	−72.78	48.29	0.20	0.25	0.37	0.08	0.42	116.54	Tributary
35,107,040	4.69	−73.05	79.90	0.04	0.32	0.38	0.04	0.58	57.04	Tributary
35,127,010	4.31	−72.08	65.08	0.14	0.56	0.35	0.03	0.51	272.39	Tributary
35,127,020	4.34	−72.16	22.10	0.22	0.51	0.38	0.50	0.38	69.56	Tributary
35,127,030	3.81	−72.29	40.41	0.16	0.83	0.32	0.03	0.51	173.87	Tributary
35,137,010	3.73	−72.37	28.00	0.09	0.58	0.42	0.06	0.50	46.24	Tributary
35,227,090	4.90	−71.44	10.91	0.15	0.32	0.52	0.28	0.33	76.90	Tributary
36,027,050	6.30	−70.19	30.99	0.25	0.13	0.54	0.25	0.21	317.32	Tributary

Parameters estimated using the least squares method based on historical streamflow records. Upstream distance measured from each station’s location along the river network.

**Table 3:** Geographic information, upstream distance, and at-station hydraulic geometry parameters for hydrological stations in the Atrato River basin.

Hydrological station code	latitude	longitude	a	b	c	f	k	m	Upstream [m]	Group river
11,027,010	5.51	−76.10	8.76	0.31	0.17	0.39	0.65	0.30	27.94	Atrato
11,027,030	5.51	−76.09	9.00	0.14	0.24	0.41	0.48	0.43	24.11	Atrato
11,027,050	5.31	−76.31	86.18	0.06	0.74	0.24	0.02	0.70	126.87	Atrato
11,027,070	5.30	−76.34	23.05	0.35	0.41	0.23	0.11	0.43	137.79	Atrato
11,047,010	5.45	−76.40	134.49	0.10	0.10	0.53	0.07	0.36	191.70	Atrato
11,047,020	5.41	−76.39	59.17	0.21	0.18	0.45	0.09	0.34	180.69	Atrato
11,057,010	6.13	−76.43	146.08	0.09	0.21	0.46	0.03	0.45	274.32	Atrato
11,077,010	6.33	−76.53	137.27	0.14	0.16	0.46	0.04	0.40	348.26	Atrato
11,017,010	5.28	−76.32	49.63	0.09	0.39	0.32	0.05	0.60	65.94	Tributary
11,027,040	5.48	−76.18	25.69	0.06	0.15	0.58	0.26	0.36	13.67	Tributary
11,037,020	5.22	−76.36	35.32	0.17	0.15	0.44	0.18	0.39	16.76	Tributary
11,047,030	5.49	−76.37	53.20	0.05	0.23	0.48	0.08	0.47	19.34	Tributary

Parameters estimated using the least squares method based on historical streamflow records. Upstream distance measured from each station’s location along the river network.

In order to compare estimated values with measured values, two hydrological monitoring stations were selected on the main channel of the Meta River: Puerto Texas (code 35,177,020), located in the upper reaches, and Aguaverde (code 35,267,080), in the lower reaches. Two stations were also included in tributaries of the upper basin: El Palmar (code 35,027,200) and La Esperanza (code 35,127,030). In the Atrato River basin, two stations were considered on the main channel: Gindrama (code 11,027,050), in the upper reaches, and Tagachi (code 11,057,010), in the lower reaches; in addition to two on tributaries: Aguasal (code 11,017,010) and Puente Las Sánchez (code 11,027,010). This selection was made in order to perform a cross-validation of the estimates of discharge, hydraulic depth, and average velocity.

For each hydrological station, the upstream distance was calculated, which is essential information for applying the multiquadratic interpolation method, which guarantees the spatial continuity of the hydraulic geometry parameters along the main channel and its tributaries. Given that these parameters are compositional in nature [6], a log-ratio transformation based on sum and product operations was applied before performing the interpolation in order to bring the data into Euclidean space. Once the interpolation was completed, the data were transformed back to the original space and closed using constraints that ensure that the product of the estimated coefficients is equal to one and that the sum of the exponents is also equal to one [11].

To optimize multiquadric interpolation, smoothing parameter values in the range of 0.1 to 0.5 were evaluated. For each configuration, the root mean square error (RMSE) was computed, and the value that minimized this error was selected for each parameter. The adjusted smoothing coefficients and corresponding RMSE values are presented in Table 4, Table 5 for the Meta and Atrato River basins, respectively.

**Table 4:** Optimal smoothing coefficients and RMSE values for hydraulic geometry parameters in the Meta River basin.

Parameter	Smoooth	RMSE
a	0.2	36.71
c	0.5	0.40
k	0.5	0.07
b	0.2	0.08
f	0.5	0.06
m	0.5	0.12

Optimal smoothing coefficients obtained by minimizing RMSE during multiquadric interpolation of hydraulic geometry parameters.

**Table 5:** Optimal smoothing coefficients and RMSE values for hydraulic geometry parameters in the Atrato River basin.

Parameter	Smoooth	RMSE
a	0.5	37.83
c	0.5	0.21
k	0.3	0.25
b	0.5	0.18
f	0.5	0.07
m	0.5	0.18

Optimal smoothing coefficients obtained by minimizing RMSE during multiquadric interpolation of hydraulic geometry parameters.

Although the coefficients a (width), c (depth), and k (velocity) showed some variability during cross-validation, a uniform smoothing value of 0.5 was adopted for all parameters in order to preserve the compositional closure of the hydraulic relationships. Table 6, Table 7 present the observed and estimated values of the hydraulic geometry coefficients at the stations selected for cross-validation, using a uniform smoothing parameter of 0.5.

**Table 6:** Observed and estimated at-station hydraulic geometry coefficients for cross-validation in the Meta River basin.

Station	a	$\hat{a}$	$e_{a}$ ( %)	c	$\hat{c}$	e_c_ ( %)	k	$\hat{k}$	e_k_ ( %)
Aguaverde	57.41	57.12	0.51	0.50	1.10	120.00	0.03	0.02	33.33
Pto_Texas	116.19	37.92	67.36	0.20	0.19	5.00	0.04	0.14	250.00
La Esperanza	40.41	27.35	32.32	0.83	0.30	63.85	0.03	0.12	300.00
El Palmar	20.55	16.59	19.27	0.21	0.29	38.09	0.23	0.20	13.04

Estimated values obtained using multiquadric interpolation with a uniform smoothing parameter of 0.5. Errors expressed as percentage differences between observed and estimated values.

**Table 7:** Observed and estimated at-station hydraulic geometry coefficients for cross-validation in the Atrato River basin.

Station	a	$\hat{a}$	$\;e_{a}$ ( %)	c	$\hat{c}$	e_c_ ( %)	k	$\hat{k}$	e_k_ ( %)
Tagachi	146.08	129.34	11.46	0.21	0.13	38.09	0.03	0.06	100.00
Gindrama	86.18	23.03	73.28	0.74	0.39	47.30	0.02	0.11	450.00
Aguasal	49.63	12.61	74.59	0.39	0.57	46.15	0.05	0.14	180.00
Pte. Sánchez	8.76	17.94	104.79	0.17	0.33	94.11	0.65	0.17	72.31

Estimated values obtained using multiquadric interpolation with a uniform smoothing parameter of 0.5. Errors expressed as percentage differences between observed and estimated values.

Table 8, Table 9 summarize the descriptive statistics of the percentage estimation errors, indicating that the model performs more reliably in the Meta River basin compared to the Atrato River basin.

**Table 8:** Descriptive statistics of percentage estimation errors for hydraulic geometry coefficients in the Meta River basin.

Statistic	a	c	k
Mean	29.87	56.74	149.09
Standard Deviation	28.20	48.57	147.04
Maximum	67.36	120.00	300.00
Minimum	0.51	5.00	13.04

Errors expressed as percentage differences between observed and estimated values for coefficients a, c, and k.

**Table 9:** Descriptive statistics of percentage estimation errors for hydraulic geometry coefficients in the Atrato River basin.

Statistic	a	c	k
Mean	66.03	56.41	200.58
Standard Deviation	39.18	25.46	172.44
Maximum	104.79	94.11	450.00
Minimum	11.46	38.09	72.31

Errors expressed as percentage differences between observed and estimated values for coefficients a, c, and k.

In general terms, the model performed better in the Meta River basin, especially for coefficient a, with average percentage errors lower than those recorded in the Atrato River basin. For coefficient c, the errors were similar in both basins, while for k, high errors were observed in both basins.

Table 10, Table 11 present the observed and estimated values of the hydraulic geometry exponents at the stations selected for cross-validation, applying a uniform smoothing parameter of 0.5 during the interpolation process.

**Table 10:** Observed and estimated at-station hydraulic geometry exponents for cross-validation in the Meta River basin.

Station	b	$\hat{b}$	e_b_ ( %)	f	$\hat{f}$	e_f_ ( %)	m	$\hat{m}$	e_m_ ( %)
Aguaverde	0.28	0.28	0.00	0.30	0.20	33.33	0.42	0.52	23.81
Pto_Texas	0.15	0.30	100.00	0.43	0.43	0.00	0.43	0.28	34.88
La Esperanza	0.16	0.22	31.50	0.32	0.39	21.87	0.51	0.39	23.53
El Palmar	0.11	0.18	63.64	0.38	0.40	5.26	0.51	0.42	17.65

Estimated values obtained using multiquadric interpolation with a uniform smoothing parameter of 0.5. Errors expressed as percentage differences between observed and estimated values for exponents b, f, and m.

**Table 11:** Observed and estimated at-station hydraulic geometry exponents for cross-validation in the Atrato River basin.

Station	b	$\hat{b}$	e_b_ ( %)	f	$\hat{f}$	e_f_ ( %)	m	$\hat{m}$	e_m_ ( %)
Tagachi	0.09	0.13	44.44	0.46	0.50	8.70	0.45	0.37	17.78
Gindrama	0.06	0.32	433.33	0.24	0.24	0.00	0.70	0.44	37.14
Aguasal	0.09	0.30	233.33	0.32	0.20	37.50	0.60	0.50	16.67
Pte. Sánchez	0.31	0.17	45.16	0.39	0.33	15.38	0.30	0.50	66.67

Estimated values obtained using multiquadric interpolation with a uniform smoothing parameter of 0.5. Errors expressed as percentage differences between observed and estimated values for exponents b, f, and m.

The results show that the model is able to reproduce with greater accuracy the exponents associated with depth (f) and, to a lesser extent, velocity (m), while the width exponent (b) shows high variability, especially in the Atrato River basin. This difference can be attributed to station density, as the Meta basin has a more extensive network that favors spatial interpolation.

Table 12, Table 13 summarize the descriptive statistics of the percentage errors for the estimated exponents. The results indicate heterogeneous performance between basins and parameters: the average error for b (width) is substantially lower in the Meta River basin, while the errors for f (hydraulic depth) and m (velocity) are considerably lower in the Atrato River basin. This suggests that the model fit is more robust for hydraulic depth and velocity in the Atrato basin and for width in the Meta basin, likely influenced by station density differences between basins.

**Table 12:** Descriptive statistics of percentage estimation errors for hydraulic geometry exponents in the Meta River basin.

Statistic	b	f	m
Mean	29.87	56.74	149.09
Standard Deviation	28.20	48.57	147.04
Maximum	67.36	120.00	300.00
Minimum	0.51	5.00	13.04

Errors expressed as percentage differences between observed and estimated values for exponents b (width), f (hydraulic depth), and m (velocity).

**Table 13:** Descriptive statistics of percentage estimation errors for hydraulic geometry exponents in the Atrato River basin.

Statistic	b	f	m
Mean	189.07	15.40	34.56
Standard Deviation	190.02	15.94	23.36
Maximum	433.33	37.50	66.67
Minimum	44.44	0.00	16.67

Errors expressed as percentage differences between observed and estimated values for exponents b (width), f (hydraulic depth), and m (velocity).

Despite the dispersion observed in the errors, the proposed approximation is acceptable considering that the estimates are derived solely from knowledge of the channel width. This finding highlights the usefulness of the method in regions with limited hydrological information, where ensuring the spatial continuity of hydraulic parameters is a priority for water resource planning and management.

Overall, the results indicate that, although the model presents systematic biases, especially in the estimation of average velocity, its compositional structure and spatial adaptability make it a suitable tool for applications in contexts with scarce data, provided that its limitations are recognized and complemented with local validations.

Scatter plots were generated comparing the observed values with the estimates for each hydraulic variable (discharge, depth, and velocity), including those obtained using the regression method. These representations correspond to the control stations selected in both basins and allow the degree of model fit to be evaluated, as well as visualizing its performance in estimating hydraulic variables based on geometric information on the width of the river measured at the gauging stations.

**Fig. 2:**
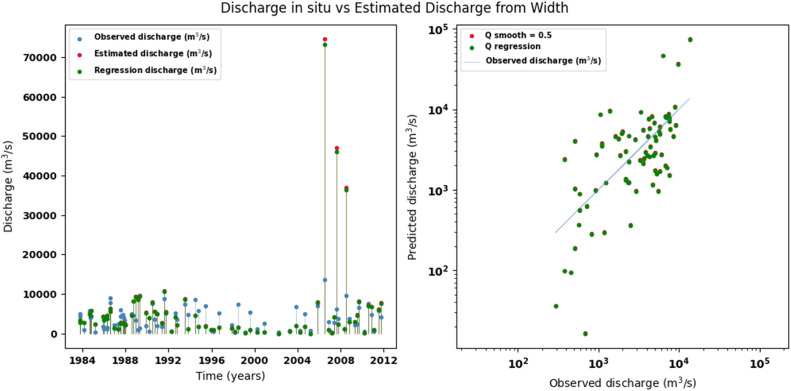
Discharge at Aguaverde station (Meta River basin).

**Fig. 3:**
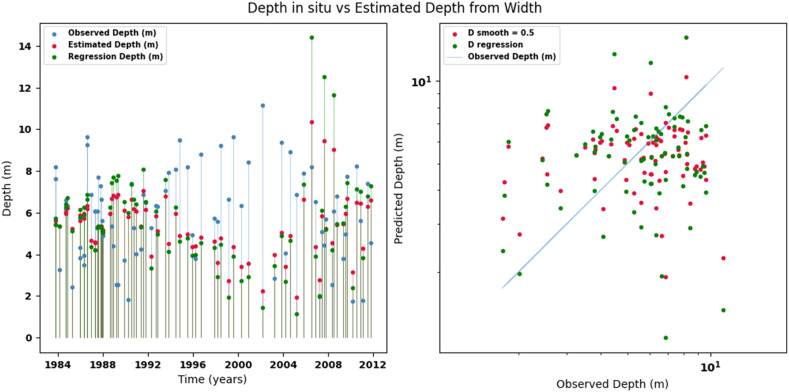
Hydraulic depth at Aguaverde station (Meta River basin).

**Fig. 4:**
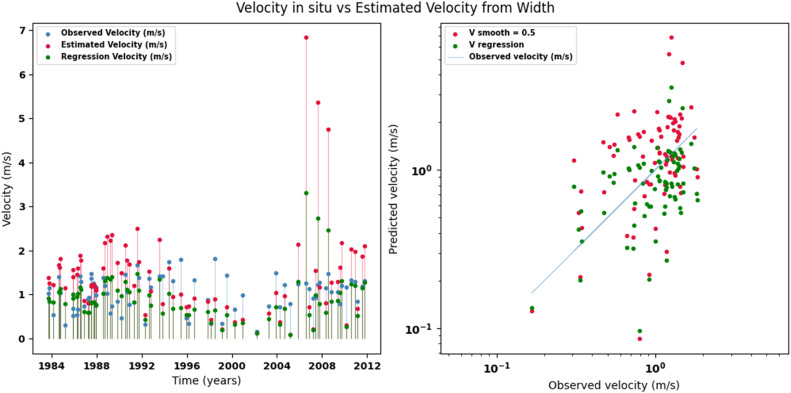
Mean velocity at Aguaverde station (Meta River basin).

**Fig. 5:**
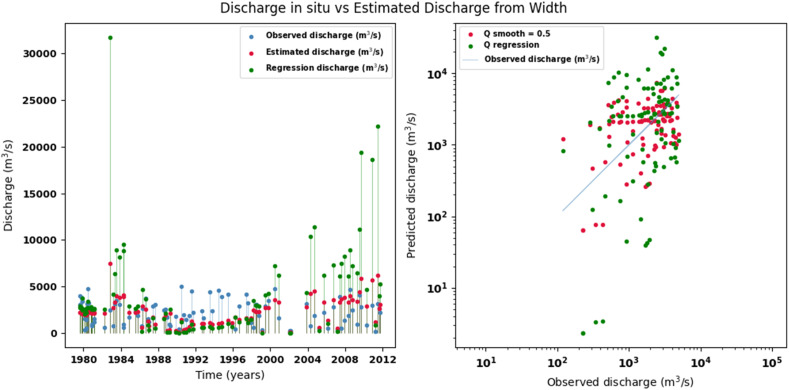
Discharge at Pto Texas station (Meta River basin).

**Fig. 6:**
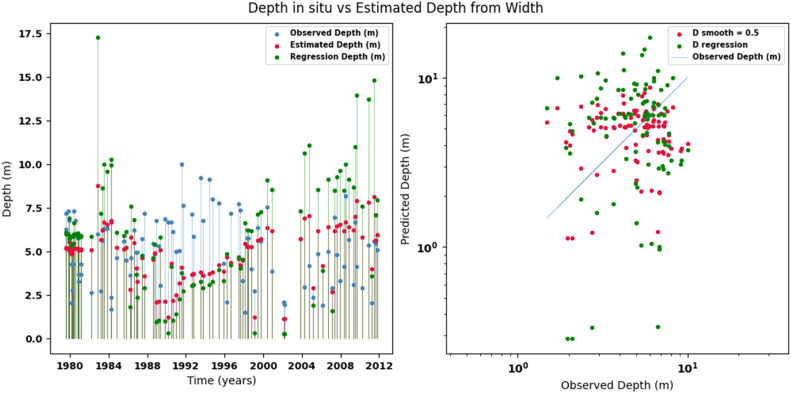
Hydraulic depth at Pto Texas station (Meta River basin).

**Fig. 7:**
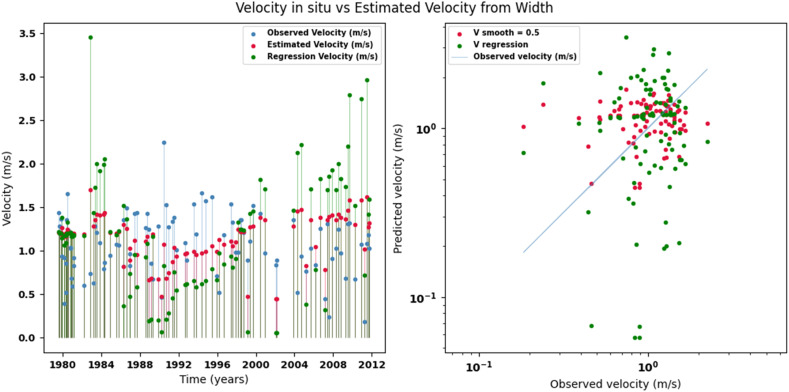
Mean velocity at Pto Texas station (Meta River basin).

**Fig. 8:**
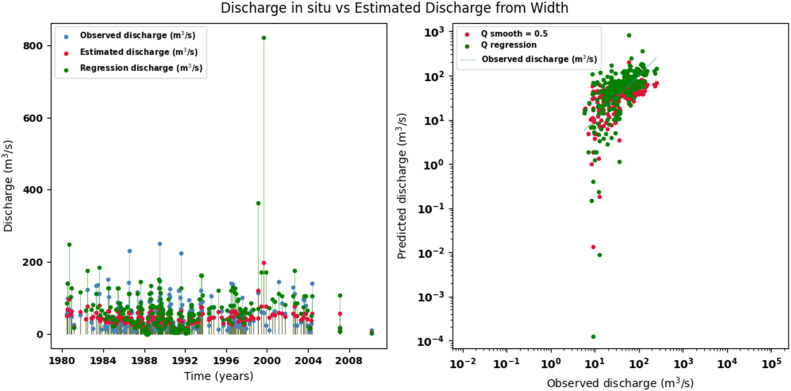
Discharge at El Palmar station (Meta River basin).

**Fig. 9:**
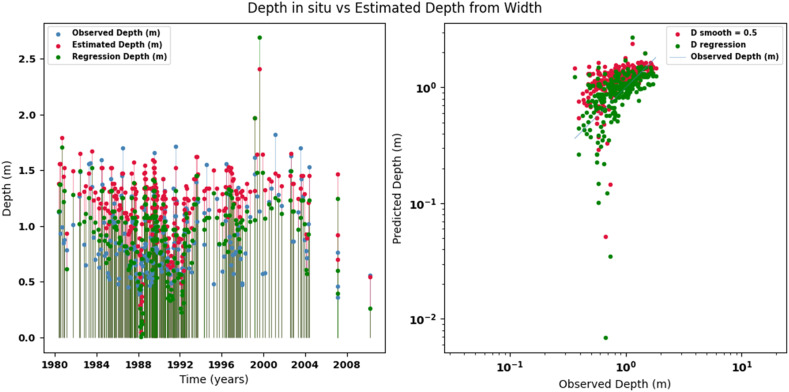
Hydraulic depth at El Palmar station (Meta River basin).

**Fig. 10:**
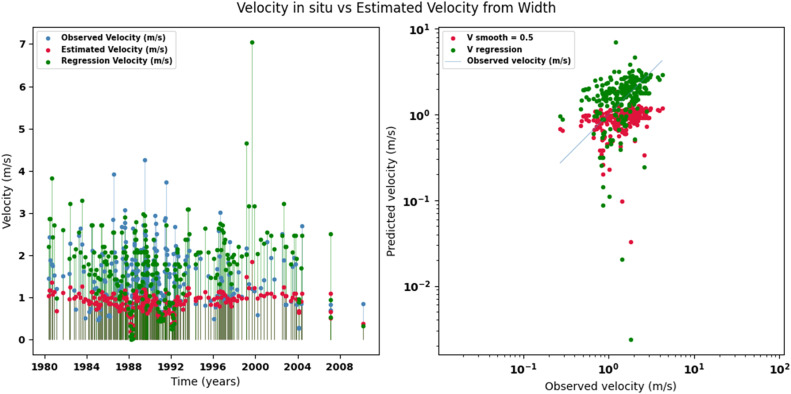
Mean velocity at El Palmar station (Meta River basin).

**Fig. 11:**
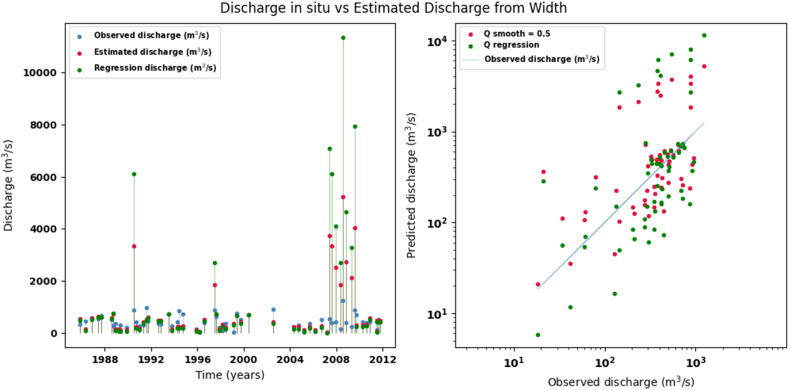
Discharge at La Esperanza station (Meta River basin).

**Fig. 12:**
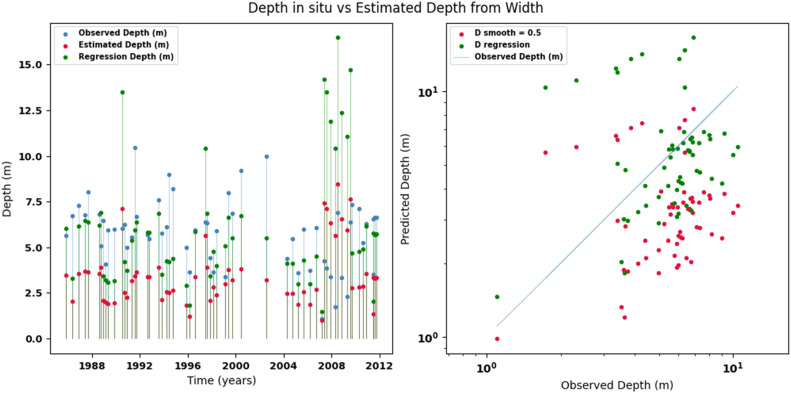
Hydraulic depth at La Esperanza station (Meta River basin).

**Fig. 13:**
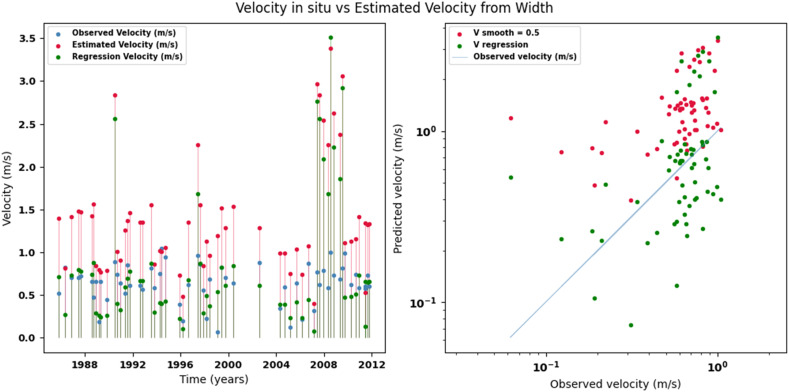
Mean velocity at La Esperanza station (Meta River basin).

Fig. 2, Fig. 3, Fig. 4, Fig. 5, Fig. 6, Fig. 7, Fig. 8, Fig. 9, Fig. 10, Fig. 11, Fig. 12, Fig. 13 illustrate the comparison between observed and estimated values of discharge, hydraulic depth, and mean velocity at four control stations in the Meta River basin (Aguaverde, Pto Texas, El Palmar, and La Esperanza). For each variable, the left panel shows an irregular time series of observed values (blue), estimates obtained by interpolation (red), and estimates obtained by regression (green). The right panel presents a log-scale scatter plot comparing observed and estimated values, highlighting the performance of the proposed SHGI method for estimating hydraulic variables from geometric information.

In addition to scatter plots, quantitative metrics were calculated to evaluate the performance of the SHGI model in estimating discharge (Q), hydraulic depth (D), and mean velocity (V). Table 14 summarizes the mean absolute percentage error (MAPE) values at four monitoring stations in the Meta River basin, comparing two approaches: multiquadric interpolation and at-station hydraulic geometry (regression).

**Table 14:** Mean absolute percentage error (MAPE) for discharge, hydraulic depth, and mean velocity at four stations in the Meta River basin.

	MAPE – Interpolation			MAPE – Hydraulic Geometry		
Station	Q	D	V	Q	D	V
Aguaverde	1.06	0.41	0.71	1.04	0.45	0.41
Pto Texas	1.04	0.47	0.41	2.02	0.76	0.71
El Palmar	0.58	0.48	0.42	0.98	0.29	0.55
La Esperanza	1.59	0.56	1.6	2.30	0.60	0.77

MAPE values compare multiquadric interpolation and at-station hydraulic geometry (regression) for discharge (Q), hydraulic depth (D), and mean velocity (V).

The results show that multiquadratic interpolation offers a more consistent fit in estimating discharge (Q) and average velocity (V), with lower MAPE values at most stations. For hydraulic depth (D), both methods present similar errors, although the proposed method (SHGI) tends to reduce variability compared to station hydraulic geometry (regression). These findings reinforce the usefulness of the interpolated approach to improve spatial continuity and accuracy in regions with limited information.

**Fig. 14:**
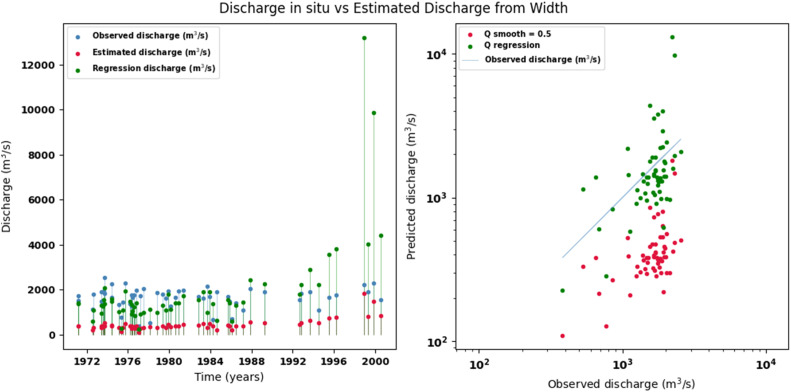
Discharge at Tagachi station (Atrato River basin).

**Fig. 15:**
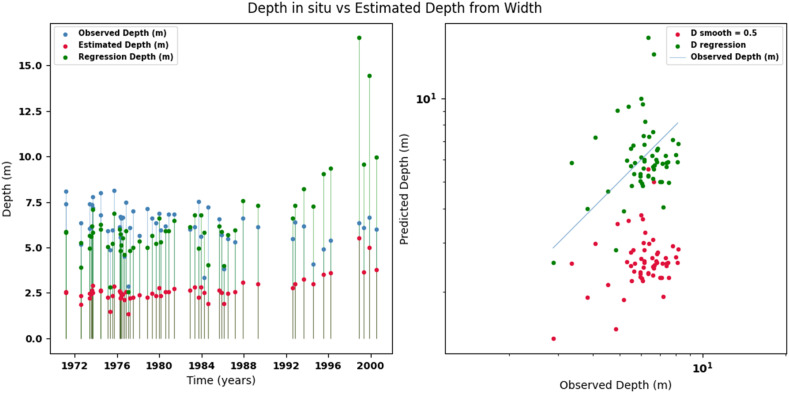
Hydraulic depth at Tagachi station (Atrato River basin).

**Fig. 16:**
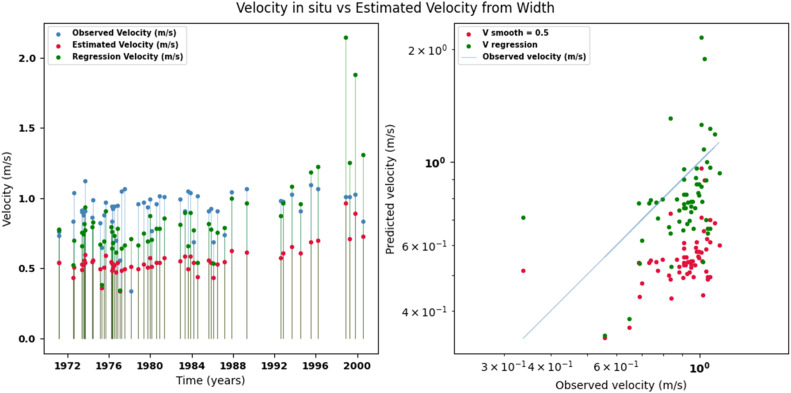
Mean velocity at Tagachi station (Atrato River basin).

**Fig. 17:**
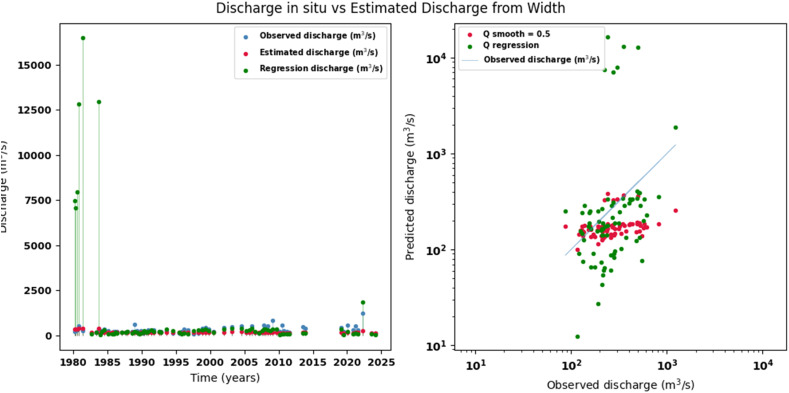
Discharge at Gindrama station (Atrato River basin).

**Fig. 18:**
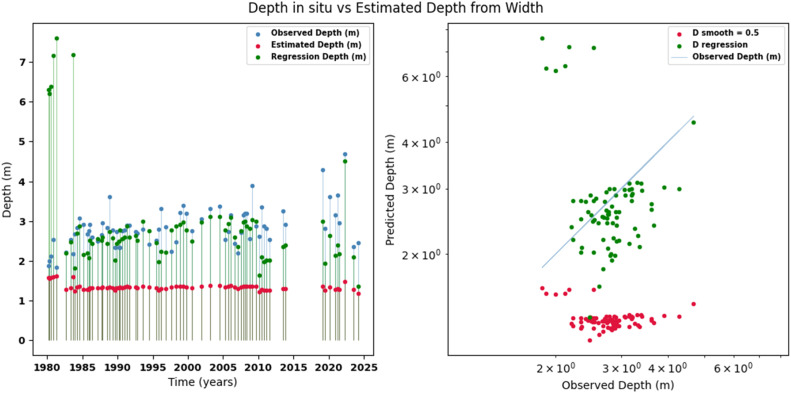
Hydraulic depth at Gindrama station (Atrato River basin).

**Fig. 19:**
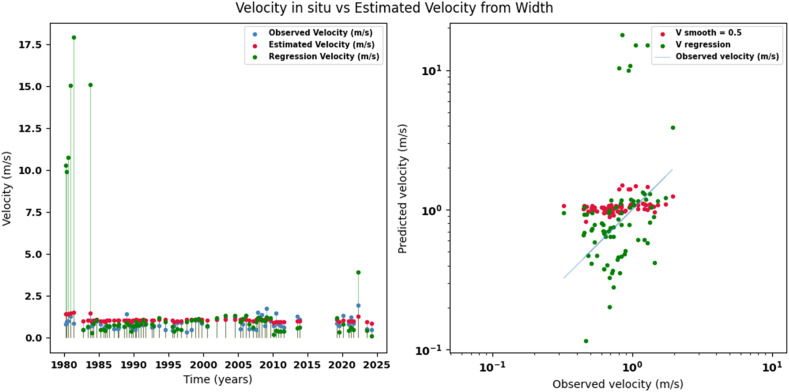
Mean velocity at Gindrama station (Atrato River basin).

**Fig. 20:**
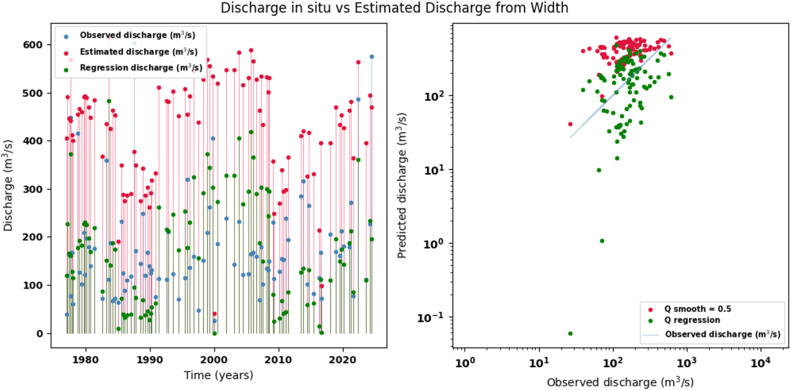
Discharge at Aguasal station (Atrato River basin).

**Fig. 21:**
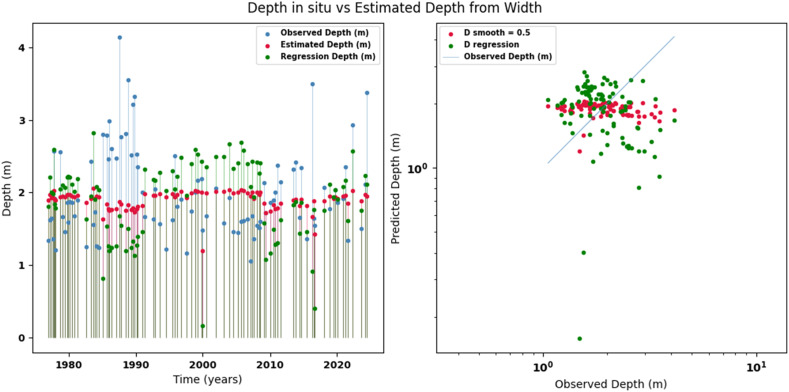
Hydraulic depth at Aguasal station (Atrato River basin).

**Fig. 22:**
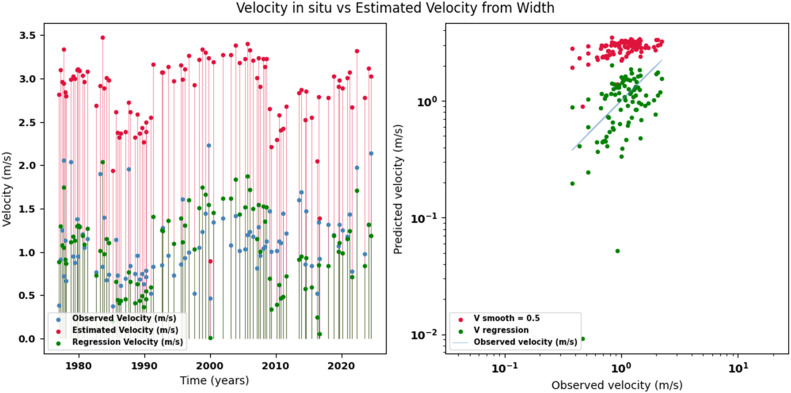
Mean velocity at Gindrama station (Atrato River basin).

**Fig. 23:**
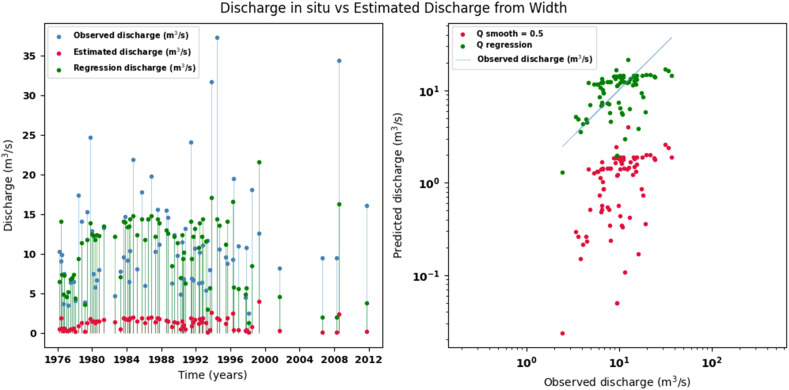
Discharge at Pte Las Sánchez station (Atrato River basin).

**Fig. 24:**
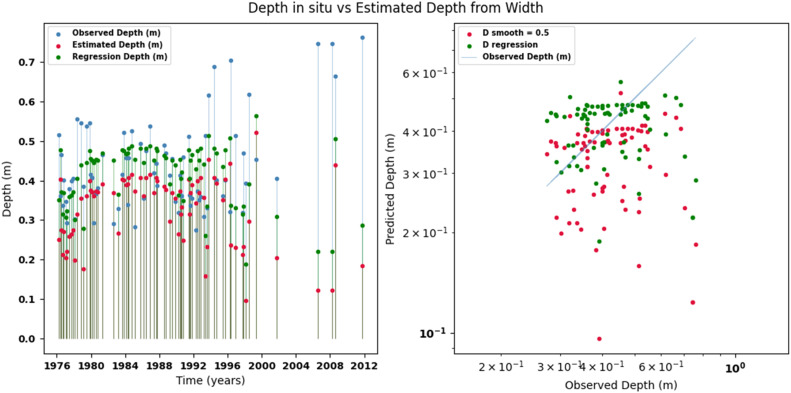
Hydraulic depth at Pte Las Sánchez station (Atrato River basin).

**Fig. 25:**
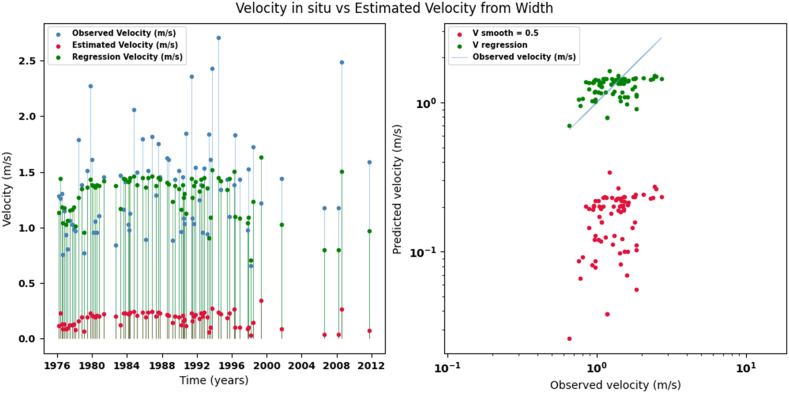
Mean velocity at Pte Las Sánchez station (Atrato River basin).

Fig. 14, Fig. 15, Fig. 16, Fig. 17, Fig. 18, Fig. 19, Fig. 20, Fig. 21, Fig. 22, Fig. 23, Fig. 24, Fig. 25 illustrate the comparison between observed and estimated values of discharge, hydraulic depth, and mean velocity at four control stations in the Atrato River basin (Tagachi, Gindrama, Aguasal, and Pte. Las Sánchez). For each variable, the left panel shows an irregular time series of observed values (blue), estimates obtained by multiquadric interpolation (red), and estimates obtained by regression (green). The right panel presents a log-scale scatter plot comparing observed and estimated values, highlighting the performance of the proposed SHGI method for reconstructing hydraulic variables from geometric information under irregular observation conditions.

In addition to scatter plots, quantitative metrics were calculated to evaluate the performance of the model (SHGI) in estimating discharge (Q), hydraulic depth (D), and average velocity (V). Table 15 summarizes the mean absolute percentage error (MAPE) values at four monitoring stations in the Atrato River basin, comparing two approaches: multiquadratic interpolation and station hydraulic geometry (regression).

**Table 15:** Mean absolute percentage error (MAPE) for discharge (Q), Hydraulic depth (D), and mean velocity (V) at four monitoring stations in the Atrato River Basin.

	MAPE – Interpolation			MAPE – Hydraulic Geometry		
Station	Q	D	V	Q	D	V
Tagachi	0.72	0.56	0.39	0.51	0.26	0.23
Gindrama	0.38	0.51	0.50	3.23	0.32	1.36
Aguasal	2.09	0.24	1.90	0.71	0.37	0.37
Pte. Sánchez	0.88	0.27	0.87	0.42	0.22	0.23

MAPE values compare multiquadric interpolation and at-station hydraulic geometry (regression) for discharge (Q), hydraulic depth (D), and mean velocity (V).

In the Atrato River basin, multiquadric interpolation did not show substantial improvements over station-based hydraulic geometry. At most stations, the mean absolute percentage error (MAPE) increased for discharge (Q) and mean velocity (V), as observed in Aguasal (Q: 0.71 % → 2.09 %, V: 0.37 % → 1.90 %) and Pte. Sánchez (Q: 0.42 % → 0.88 %, V: 0.23 % → 0.87 %). Only Gindrama exhibited a reduction in velocity error (1.36 % → 0.50 %), which represents an exception. These results suggest that the effectiveness of the SHGI method is strongly influenced by station density and spatial heterogeneity, limiting its performance in scenarios with low data availability.

The difference in the performance of the SHGI method between the two basins can be largely attributed to the density and representativeness of the available information. In the Meta River basin, the estimation relied on 44 hydrological stations distributed throughout the basin, providing a robust basis for interpolation and reducing uncertainty.

In contrast, the Atrato River basin had only nine stations, limiting the method’s ability to capture spatial and hydrological variability. This constraint, combined with geographical, hydrological, edaphological, and geological differences between the two basins, explains the lower performance observed in the Atrato. Nevertheless, the results obtained are acceptable considering that the estimation is based solely on one variable channel width (W) without additional information that would allow for a more complex statistical adjustment.

## Limitations

Although the SHGI method demonstrates potential for estimating hydraulic variables from geometric information, its application is subject to several constraints that must be acknowledged to ensure reliable interpretation and future improvements.

Despite the promising results obtained through spatial interpolation of station-based hydraulic geometry parameters, the SHGI method presents certain limitations that should be considered:

1. **Sensitivity in tributary zones:** Model accuracy decreases in areas influenced by tributaries, where empirical relationships between hydraulic and geometric variables are weaker, resulting in higher relative errors in the estimation of mean velocity and hydraulic depth.
2. **Dependence on historical data:** The application of the method requires historical discharge records at stations located upstream and downstream of the point of interest. In regions lacking such information, the estimation capability is significantly constrained.
3. **Coefficient variability:** The coefficients a, *c*, and *k* exhibit high variability and sensitivity to calibration, which may affect model stability under different hydrological conditions.
4. **Compositional transformation:** The need to apply log-ratio transformations to preserve the compositional nature of the parameters introduces additional complexity in implementation and analysis.
5. **Limitations in mean velocity estimation:** Mean velocity is the variable with the greatest dispersion and error in the estimates, suggesting that the model does not fully capture its spatial behavior.
6. **Scalability and station density:** Results from the Meta and Atrato basins show that method performance decreases significantly when station density is low, which is expected in processes that estimate multiple hydraulic variables from a single geometric variable (channel width, W). This limitation underscores the need for a minimum control network to ensure reliable interpolation.

Overall, these findings indicate that, although the SHGI model exhibits systematic biases particularly in mean velocity estimation its compositional structure and spatial adaptability make it suitable for applications in regions with limited information, provided that its constraints are acknowledged and complemented with field-based geomorphological and biophysical evidence, such as historical water level marks, hydromorphic soils, or vegetation indicative of wet environments.

Future research should focus on reducing these limitations by incorporating additional geomorphic and hydrological predictors, improving station coverage, and exploring hybrid approaches that combine spatial interpolation with machine learning techniques to enhance accuracy and scalability.

## Ethics statements

The method discussed in this scientific article did not involve studies with living beings.

## CRediT authorship contribution statement

**Eduardo Zamudio-Huertas:** Investigation, Validation, Methodology, Supervision, Software, Formal analysis, Writing – original draft, Writing – review & editing. **César Augusto García-Ubaque:** Data curation, Formal analysis, Writing – review & editing. **Nelson Obregón-Neira:** Validation, Methodology, Conceptualization, Data curation, Formal analysis, Writing – review & editing.

## Declaration of competing interest

The authors declare that they have no known competing financial interests or personal relationships that could have appeared to influence the work reported in this paper.

## Data Availability

Data will be made available on request.
